# 

**Published:** 1893-09

**Authors:** 


### CONTENTS.

ORIGINAL ARTICLES—

Ante and Retro—Positions of the Uterus—Their Pathology, Symtomatoly and treatment. (Illustrated.) By W. W. Stewart, M. D., Columbus, Ga.......... 457

Large Cyst of Kidney—Nephrectomy—Recovery. By L. S. McMurtry, M. D., Louisville, Ky.......... 465

Osteo-Sarcoma of the Femur—Recovery and Death. By C. A. White, M. D., Atlanta, Ga.......... 470

Treatment of Obesity and Fatty Degeneration, By G. H. Thompson, A. M., M. D., St. Louis, Mo.......... 472

SOCIETY REPORTS—

Philadelphia Academy of Surgery.......... 476

EDITORIALS—

Abdominal Surgery by Untrained Operators with Deficient Appliances.......... 499

Proposed Organization of Medical Journal Publishers.......... 500

Delegates from Georgia to the Pan-American Medical Congress 501

EDITORIAL NOTES.......... 502–504

(Continued on Next Page.)

BOOK REVIEWS—

A System of Genito-Urinary Diseases; Syphiology and Dermatology, edited by P. A. Morrow, M. D.; Sterility in the Woman and its Treatment, by Dr. De Sinety; Missouri State Medical Directory.......... 485

SELECTIONS AND ABSTRACTS—

Hypnotism in Russsia.......... 475
In the Treatment of Choleraic Diarrhœa.......... 484
The Punishment for the Crime of Producing Abortions.......... 486
The Clinical Application of Ingluvin.......... 487
Period of Infectiousness of Contagious Diseases.......... 489
Diarrhœa in Children.......... 490
The Liquor Habit.......... 491
Puerperal Septicæmia.......... 495
Nitrate of Strychnine in Alcoholism.......... 496
The Successful Treatment of Pneumonia with Oxygen.......... 496
The Treatment of Post-Partum Hemorrhage.......... 497
The Treatment of Eclampsia.......... 497
Laugh.......... 498
Strontium Bromide in Epilepsy.......... 510
Painful Pregnancies and Sub-Involution.......... 512

PRESCRIPTION DEPARTMENT—.......... 506–507
SPECIAL NOTES— 508–514

